# Signal Separation Operator Based on Wavelet Transform for Non-Stationary Signal Decomposition

**DOI:** 10.3390/s24186026

**Published:** 2024-09-18

**Authors:** Ningning Han, Yongzhen Pei, Zhanjie Song

**Affiliations:** 1 School of Mathematical Sciences, Tiangong University, Tianjin 300387, China; ningninghan@tiangong.edu.cn (N.H.); yzhpei@tiangong.edu.cn (Y.P.); 2 Geogia Tech Shenzhen Institute, Tianjin University, Shenzhen 518055, China

**Keywords:** non-stationary signal separation, instantaneous frequency, continuous wavelet transform

## Abstract

This paper develops a new frame for non-stationary signal separation, which is a combination of wavelet transform, clustering strategy and local maximum approximation. We provide a rigorous mathematical theoretical analysis and prove that the proposed algorithm can estimate instantaneous frequencies and sub-signal modes from a blind source signal. The error bounds for instantaneous frequency estimation and sub-signal recovery are provided. Numerical experiments on synthetic and real data demonstrate the effectiveness and efficiency of the proposed algorithm. Our method based on wavelet transform can be extended to other time–frequency transforms, which provides a new perspective of time–frequency analysis tools in solving the non-stationary signal separation problem.

## 1. Introduction

Many signals that occur in a wide range of engineering and scientific fields, such as remote sensing signals, mobile communication, sensor arrays, radar systems, are composed of nonlinear and non-stationary components. Decomposition of such signals and efficient extraction of information from individual components including frequencies, amplitudes and waveforms will be useful to identify and quantify the underlying multicomponent signal. In the last few decades, many decomposition methods have been developed to deal with nonlinear and non-stationary systems in time, frequency or time–frequency domains. In this paper, we model a non-stationary signal by

(1) $$f\left(t\right)=\sum_{k=1}^{K}A_{k}\left(t\right)\cos\left(2\pi \phi_{k}\left(t\right)\right),$$

where the phase functions $\phi_{k}\left(t\right)$ are differentiable, and the amplitude functions $A_{k}\left(t\right)$ are non-negative and continuous.

The empirical mode decomposition (EMD) proposed in [1] is an iterative algorithm that decomposes the given non-stationary signal into a sum of intrinsic mode functions (IMFs) with a minimally oscillatory function, called trend, as the remainder and computes the instantaneous frequency on a time interval of each IMF by applying the Hilbert transform.

Although more subsequent studies, like [2,3,4,5,6,7,8], have been proposed to improve and extend EMD, there is no rigorous mathematical analysis at this moment. Inspired by EMD and compressed sensing theory, the authors in [9] introduce a nonlinear matching pursuit method (NMP) to find the sparsest representation of a signal over the possible largest dictionary, consisting of intrinsic mode functions with the form {a(t)cos(θ(t))}. As an EMD-like tool, the empirical wavelet transform [10] decomposes a given signal into different modes by designing an adaptive wavelet basis. Additionally, variational model decomposition [11] is proposed to decompose a signal into its principal modes with specific sparsity properties. Note that variational model decomposition is based on the assumption of narrow-band characteristics; an alternative algorithm named variational nonlinear chirp mode decomposition [12] is developed to concurrently extract all modes. The other similar strategy is adaptive local iterative filtering [13,14], which employs an adaptive iterative filtering to decompose nonlinear and non-stationary signals. To deal with oscillatory signals composed of different modes with fast-varying instantaneous frequencies, a convex optimization [15] is proposed to achieve the decomposition.

The reassignment method [16,17,18,19,20] and the synchrosqueezing transform (SST) [21,22] (a special reassignment technique) are other well-known time–frequency analysis tools. The mathematical theory of SST based on continuous wavelet transform (CWTSST) is presented in [22]. The main idea of SST is to concentrate the time–frequency representation into the frequency re-assignment reference. In this way, it will sharpen the time–frequency representation and decrease smearing while still reconstructing different signal components. As powerful tools for the mode retrieval, SST can be extended to other time–frequency representations, such as wavelet packet transform [23], curvelet transform [24], S-transform [25] and short-time Fourier transform [26,27]. The stability and theoretical properties of SST have been studied extensively [28,29,30,31]. For obtaining a more accurate estimate of instantaneous frequencies than the original SST, two-order or high-order adaptive SST [32,33,34,35,36,37,38] have been put forward to achieve mode retrieval results with a high accuracy.

Recently, an effective non-stationary signal separation method based on discrete modulated short-time Fourier transform was introduced in [39]. The procedure is first to remove (or extract) the polynomial trend from the blind source signal. The second step is to apply the signal separation operator to some discrete samples in the fixed time and extract different modes and instantaneous frequencies via finding the maximum over the frequency domain. In this regard, a family of minimum-supported cardinal spline-wavelets is introduced and developed in [40]. Unfortunately, its rigorous mathematical theoretical analysis of the error bounds for instantaneous frequency estimation and sub-signal recovery is still an open problem. It is important to point out that the objective of [40] is the implementation of non-stationary signal separation by a special wavelet. The extension of non-stationary signal separation to generalized continuous wavelet transform, (CWT) along with rigorous mathematical theoretical analysis, deserves further study.

To solve this problem, in this paper, we introduce a direct, local and mathematical rigorous non-stationary signal separation operator based on wavelet transform (CWTSSO) for analyzing and decomposing non-stationary data. Considering a general adaptive harmonic model

(2) $$f\left(t\right)=\sum_{k=1}^{K}A_{k}\left(t\right)e^{i2\pi \phi_{k}\left(t\right)},$$

where the phase functions $\phi_{k}\left(t\right)$ are differential, and the amplitude functions $A_{k}\left(t\right)$ are non-negative and continuous. Observe that the non-stationary signal model (1) can be regarded as the real part of adaptive harmonic model. Let $\psi_{\alpha }$ be a continuous wavelet and f(t) be a given signal. The CWT of f(t) is defined by

(3) $$\begin{array}{c} \left(W_{\psi_{\alpha }}f\right)\left(t,a\right)=\frac{1}{a}\int_{-\infty }^{+\infty }\psi_{\alpha }\left(\frac{x-t}{a}\right)f\left(x\right)dx, \end{array}$$

where $\psi_{\alpha }\left(x\right)=\alpha \psi \left(\alpha x\right)$ and the parameter α controls the width of the wavelet. Then, $\left(W_{\psi_{\alpha }}f\right)\left(t,a\right)$ can be used directly to extract all instantaneous frequencies and reconstruct all modes by achieving energy-concentrated clusters with an approximate threshold and finding the maximum over scales for each cluster. The superiority of CWTSSO is a more simple and direct decomposition method that reconstructs all instantaneous frequencies and modes simultaneously. It is different from CWTSST [22], which is to first compute a reference frequency value from CWT of the given signal and then to apply this value for SST to further obtain estimates of all instantaneous frequencies and modes. Compared with CWTSST, CWTSSO does not require the synchrosqueezing transform to compute reference frequencies as required by CWTSST. Hence, the CWTSSO provides superior efficiency against the CWTSST, as illustrated in numerical experiments (see Table 1 in Section 3). It should be pointed out that the proposed time–frequency analysis tool can be extended to other time–frequency transforms, e.g., wavelet packet transform, curvelet transform, S-transform, short-time Fourier transform, etc. This paper provides a new perspective of time–frequency analysis tools in solving the non-stationary signal separation problem.

## 2. Main Result

To facilitate the proof of the main result to be stated and established in this section, we consider a class of wavelet functions with certain conditions on the amplitude and phase functions that allow us to separate signal components, to compute instantaneous frequencies (IFs) and to obtain both instantaneous amplitudes (IAs) and signal components.

**Definition 1.**  *Let $A_{\alpha }$ denote the set consisting of general adaptive harmonic models defined by*

(4) $$f\left(t\right)=\sum_{\ell =1}^{K}f_{\ell }\left(t\right)=\sum_{\ell =1}^{K}A_{\ell }\left(t\right)e^{i2\pi \phi_{\ell }\left(t\right)}$$

*with*

(5) $$\begin{array}{cc} \; & M=M\left(t\right):=\sum_{\ell =1}^{K}|A_{\ell }\left(t\right)|,\mu =\mu \left(t\right):=\min_{1\leq \ell \leq K}\left|A_{\ell }\left(t\right)\right|,\\ \; & B=B\left(t\right):=\max_{1\leq \ell \leq K}|\phi_{\ell }^{\prime }\left(t\right)|,\nu =\nu \left(t\right):=\min_{1\leq \ell \leq K}\left|\phi_{\ell }^{\prime }\left(t\right)\right|, \end{array}$$

*and there exists α=α(t)>0, such that*

(6) $$\begin{array}{cc} \; & |A_{\ell }\left(t+u\right)-A_{\ell }\left(t\right)|\leq \alpha^{2}\left|u|\right|A_{\ell }\left(t\right)|,\\ \; & |\phi_{\ell }^{\prime }\left(t+u\right)-\phi_{\ell }^{\prime }\left(t\right)|\leq \alpha^{3}\left|u|\right|\phi_{\ell }^{\prime }\left(t\right)|,\;\;\;\ell =1,\ldots ,K. \end{array}$$

*In addition, we assume that the IFs satisfy*

(7) $$\begin{array}{c} \min_{1\leq k\neq \ell \leq K}\left|\frac{1}{\phi_{\ell }^{\prime }\left(t\right)}-\frac{1}{\phi_{k}^{\prime }\left(t\right)}\right|=:\eta \left(t\right)=\eta >0. \end{array}$$

The main result of this paper is as follows:

**Theorem 1.**  *Let $f\left(t\right)\in A_{\alpha }$ for some α>0. Let ψ be a wavelet such that its Fourier transform $\hat{\psi }$ is supported in [κ/α−Δ,κ/α+Δ] and $\rho =\max|\hat{\psi }\left(\cdot \right)|$=$|\hat{\psi }\left(\kappa /\alpha \right)|$. Consider the continuous wavelet transform $\left(W_{\psi_{\alpha }}f\right)\left(t,a\right)$, as defined in (3), with the set*

$$\begin{array}{c} S=S\left(t\right):=\{a=a\left(t\right):|\left(W_{\psi_{\alpha }}f\right)\left(t,a\right)|\geq \frac{\mu \rho }{2}\} \end{array}$$

*and*

(8) $$\begin{array}{c} \alpha <\min\{\frac{\eta \kappa \upsilon }{2\Delta },\frac{\rho \mu }{2\left(aMI_{1}+2\pi MBI_{2}a^{2}\right)},\frac{\rho \mu \nu^{2}}{4(\nu \kappa MI_{1}+2\pi MBI_{2}\kappa^{2})}\}, \end{array}$$

*where a∈S. The following statements hold:*

*(a)*  *The set S can be expressed as a disjoint union of exactly K non-empty sets, i.e.,*
(9) $$S_{\ell }=S_{\ell }\left(t\right):=\{a\in S\left(t\right):|a-\frac{\kappa }{\phi_{\ell }^{\prime }\left(t\right)}\left|\leq \frac{\Delta \alpha }{\nu }\right\},\;\;\ell =1,\cdots ,K.$$
*(b)*  *Let*
$${\overset{ˇ}{a}}_{\ell }={\overset{ˇ}{a}}_{\ell }\left(t\right):=\arg\max_{a\in S_{\ell }}\left|\left(W_{\psi_{\alpha }}f\right)\left(t,a\right)\right|,\;\;\ell =1,\cdots ,K,$$
  *then*
(10) $$\begin{array}{cc} \; & \left|\right|\left(W_{\psi_{\alpha }}f\right)\left(t,{\overset{ˇ}{a}}_{\ell }\right)\left|/\rho -\right|A_{\ell }\left(t\right)\left|\right|\\ \; & \leq \alpha^{3}2\pi MBI_{2}\Delta^{2}/\left(\rho \nu^{2}\right)+\alpha^{2}\left(4\pi MBI_{2}\Delta \kappa /\left(\rho \nu^{2}\right)+MI_{1}\Delta /\left(\rho \nu \right)\right)\\ \; & +\alpha \left(MI_{1}\kappa /\left(\rho \nu \right)+2\pi MBI_{2}\kappa^{2}/\left(\rho \nu^{2}\right)\right), \end{array}$$
(11) $$\begin{array}{c} |{\overset{ˇ}{a}}_{\ell }-\frac{\kappa }{\phi_{\ell }^{\prime }\left(t\right)}|=\alpha o\left(1\right), \end{array}$$
  *and*
(12) $$\begin{array}{cc} \; & |\left(W_{\psi_{\alpha }}f\right)\left(t,{\overset{ˇ}{a}}_{\ell }\right)/\rho -A_{\ell }\left(t\right)e^{i2\pi \phi_{\ell }\left(t\right)}|\\ \; & \leq \alpha^{3}2\pi MBI_{2}\Delta^{2}/\left(\rho \nu^{2}\right)+\alpha^{2}\left(4\pi MBI_{2}\Delta \kappa /\left(\rho \nu^{2}\right)+MI_{1}\Delta /\left(\rho \nu \right)\right)\\ \; & +\alpha (MI_{1}\kappa /\left(\rho \nu \right)+2\pi MBI_{2}\kappa^{2}/\left(\rho \nu^{2}\right)+2\pi MBI_{0}o\left(1\right)/\rho ) \end{array}$$
  *as $\alpha \to 0^{+}$.*

**Figure 1:**
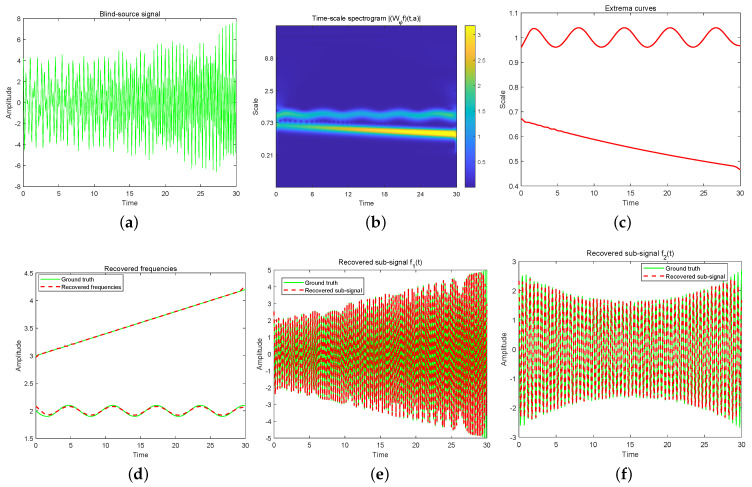
(**a**) Blind−source signal f(t). (**b**) Time-scale spectrogram $|\left(W_{\psi }f\right)\left(t,a\right)|$. (**c**) Extrema scale curves. (**d**) Recovered frequencies. (**e**) Recovered sub-signal $f_{1}\left(t\right)$. (**f**) Recovered sub-signal $f_{2}\left(t\right)$.

Before proceeding to the theoretical proof, we would like to explain the implementation of the proposed method by a numerical example. To illustrate the process, we consider the following multicomponent signal,

$$\left\{\begin{array}{l} \begin{array}{c} f\left(t\right)=f_{1}\left(t\right)+f_{2}\left(t\right),\;\;0\leq t\leq 30,\\ f_{1}\left(t\right)=\left(t/10+2\right)\cos\left(2\pi (3t+t^{2}/50)\right),\\ f_{2}\left(t\right)=\exp\left(t^{2}/450-t/15+1\right)\cos\left(2\pi (2t+\cos(t)/10)\right), \end{array} \end{array}\right.$$

as shown in Figure 1a. This experiment is carried out using Morlet wavelet, defined by

$$\begin{array}{c} \hat{\psi }\left(\xi \right)=k_{0}\sigma \sqrt{2\pi }\left(e^{-\frac{1}{2}\sigma^{2}{\left(\kappa -\xi \right)}^{2}}-e^{-\frac{1}{2}\sigma^{2}\left(\kappa^{2}+\xi^{2}\right)}\right), \end{array}$$

where $k_{0}$ is a constant, and κ and σ are wavelet parameters. With discrete grids of scales, the first step is to apply discrete wavelet transform to uniform or nonuniform samples $f(t_{n})$, n=1,2,⋯,N, where *N* is the number of samples and $\left\{t_{n}\right\}$ denotes uniform or nonuniform sampling points. As shown in the time-scale spectrogram $|\left(W_{\psi }f\right)\left(t,a\right)|$ (see Figure 1b), we can obtain two narrow bands (clusters) after thresholding. The second step is to find two extrema scale curves ${\overset{ˇ}{a}}_{\ell }\left(t\right)$ (ℓ=1,2) in the narrow bands, as shown in Figure 1c. The third step is to compute instantaneous frequencies and signal components: $\phi_{\ell }^{\prime }\left(t\right)=\kappa /{\overset{ˇ}{a}}_{\ell }\left(t\right)$, $f_{\ell }\left(t\right)=2R$e[$\left(W_{\psi }f\right)\left(t,{\overset{ˇ}{a}}_{\ell }\right)/\rho ]$, where Re denotes taking the real part of a complex number. Figure 1d–f show reconstructed results of instantaneous frequencies and sub-signals. The pseudo-code of our method can be found in Algorithm 1.
**Algorithm 1:** CWTSSO for non-stationary signal decomposition**Input:** signal f(t), μ (a small thresholding parameter).Calulate CWT of f(t) to obtain $\left(W_{\psi }f\right)\left(t,a\right)$.For each *t*, cluster $|\left(W_{\psi }f\right)\left(t,a\right)|>\frac{\mu }{2}$ to obtain precisely *K* clusters $S_{\ell }\left(t\right)$, ℓ=1,…,K.Extrema estimation ${\overset{ˇ}{a}}_{\ell }\left(t\right)=\arg\max_{a\in S_{\ell }\left(t\right)}\left|\left(W_{\psi }f\right)\left(t,a\right)\right|$.**Output:** Recovered frequencies $\phi_{\ell }^{\prime }\left(t\right)=\kappa /{\overset{ˇ}{a}}_{\ell }\left(t\right)$,                    recovered modes $f_{\ell }\left(t\right)=2R$e[$\left(W_{\psi }f\right)\left(t,{\overset{ˇ}{a}}_{\ell }\right)/\rho ]$.

We then present the proof of Theorem 1. In this proof, let

(13) $$\begin{array}{cc} \; & \left(Q_{\psi_{\alpha }}f\right)\left(t,a\right)=\sum_{\ell =1}^{K}A_{\ell }\left(t\right)e^{i2\pi \phi_{\ell }\left(t\right)}{\hat{\psi }}_{\alpha }\left(a\phi_{\ell }^{\prime }\left(t\right)\right). \end{array}$$

**Lemma 1.**  *For $f\left(t\right)\in A_{\alpha }$, let $\left(W_{\psi_{\alpha }}f\right)\left(t,a\right)$ be its continuous wavelet transform and $\left(Q_{\psi_{\alpha }}f\right)\left(t,a\right)$ be the approximation of $\left(W_{\psi_{\alpha }}f\right)\left(t,a\right)$ defined by (13), then*

(14) $$\begin{array}{c} |\left(W_{\psi_{\alpha }}f\right)\left(t,a\right)-\left(Q_{\psi_{\alpha }}f\right)\left(t,a\right)|\leq aMI_{1}\alpha +2\pi MBI_{2}a^{2}\alpha . \end{array}$$

Using the fact that

(15) $$\begin{array}{cc} \; & \left(Q_{\psi_{\alpha }}f\right)\left(t,a\right)=\sum_{\ell =1}^{K}A_{\ell }\left(t\right)e^{i2\pi \phi_{\ell }\left(t\right)}{\hat{\psi }}_{\alpha }\left(a\phi_{\ell }^{\prime }\left(t\right)\right)\\ \; & =\sum_{\ell =1}^{K}A_{\ell }\left(t\right)e^{i2\pi \phi_{\ell }\left(t\right)}\int \frac{\alpha }{a}\psi \left(\frac{\alpha (x-t)}{a}\right)e^{i2\pi \left(x-t\right)\phi_{\ell }^{\prime }\left(t\right)}dx, \end{array}$$

together with (5) and (6), we have

(16) $$\begin{array}{cc} \; & |\left(W_{\psi_{\alpha }}f\right)\left(t,a\right)-\left(Q_{\psi_{\alpha }}f\right)\left(t,a\right)|\\ \; & \leq \sum_{\ell =1}^{K}\int \frac{\alpha }{a}\psi \left(\frac{\alpha (x-t)}{a}\right)\left|A_{\ell }\left(x\right)-A_{\ell }\left(t\right)\right|e^{i2\pi \phi_{\ell }\left(x\right)}dx\\ \; & +\sum_{\ell =1}^{K}\int A_{\ell }\left(t\right)\left|e^{i2\pi \phi_{\ell }\left(x\right)}-e^{i2\pi \phi_{\ell }\left(t\right)}e^{i2\pi \left(x-t\right)\phi_{\ell }^{\prime }\left(t\right)}\right|\frac{\alpha }{a}\psi \left(\frac{\alpha (x-t)}{a}\right)dx\\ \; & \leq \sum_{\ell =1}^{K}\int \frac{\alpha }{a}\psi \left(\frac{\alpha (x-t)}{a}\right)\alpha^{2}|A_{\ell }\left(t\right)||x-t|dx\\ \; & +\sum_{\ell =1}^{K}\int A_{\ell }\left(t\right)2\pi \left|\phi_{\ell }\left(x\right)-\phi_{\ell }\left(t\right)-\left(x-t\right)\phi_{\ell }^{\prime }\left(t\right)\right|\frac{\alpha }{a}\psi \left(\frac{\alpha (x-t)}{a}\right)dx\\ \; & \leq aMI_{1}\alpha +\sum_{\ell =1}^{K}\int A_{\ell }\left(t\right)2\pi |\left(x-t\right)||\phi_{\ell }^{\prime }\left(t\right)-\phi_{\ell }^{\prime }\left(\zeta \right)|\frac{\alpha }{a}\psi \left(\frac{\alpha (x-t)}{a}\right)dx\\ \; & \leq aMI_{1}\alpha +\sum_{\ell =1}^{K}\int A_{\ell }{\left(t\right)2\pi |\left(x-t\right)|}^{2}\alpha^{3}\left|\phi_{\ell }^{\prime }\left(t\right)\right|\frac{\alpha }{a}\psi \left(\frac{\alpha (x-t)}{a}\right)dx\\ \; & \leq aMI_{1}\alpha +2\pi MBI_{2}a^{2}\alpha , \end{array}$$

where $I_{n}={\int |x|}^{n}\left|\psi \left(x\right)\right|dx$.

**Lemma 2.**  *For $f\left(t\right)\in A_{\alpha }$, let $\left(Q_{\psi_{\alpha }}f\right)\left(t,a\right)$ be defined in (13), then*

(17) $$\begin{array}{c} |\left(Q_{\psi_{\alpha }}f\right)\left(t,a\right)-A_{k}\left(t\right)e^{i2\pi \phi_{k}\left(t\right)}{\hat{\psi }}_{\alpha }\left(a\phi_{k}^{\prime }\left(t\right)\right)|=0,\;\;\forall a\in S_{k}\left(t\right), \end{array}$$

(18) $$\begin{array}{cc} \; & |\left(Q_{\psi_{\alpha }}f\right)\left(t,\frac{\kappa }{\phi_{k}^{\prime }\left(t\right)}\right)-\rho A_{k}\left(t\right)e^{i2\pi \phi_{k}\left(t\right)}|=0. \end{array}$$

**Proof.**  Since $\psi_{\alpha }\left(t\right)=\alpha \psi \left(\alpha t\right)$ and supp$\left(\hat{\psi }\right)\subseteq \left[\kappa /\alpha -\Delta ,\kappa /\alpha +\Delta \right]$, we have supp$\left({\hat{\psi }}_{\alpha }\right)\subseteq \left[\kappa -\Delta \alpha ,\kappa +\Delta \alpha \right]$. Indeed,

$$\begin{array}{c} |\left(Q_{\psi_{\alpha }}f\right)\left(t,a\right)-A_{k}\left(t\right)e^{i2\pi \phi_{k}\left(t\right)}{\hat{\psi }}_{\alpha }\left(a\phi_{k}^{\prime }\left(t\right)\right)\left|=\right|\sum_{\ell \neq k}^{K}A_{\ell }\left(t\right)e^{i2\pi \phi_{\ell }\left(t\right)}{\hat{\psi }}_{\alpha }\left(a\phi_{\ell }^{\prime }\left(t\right)\right|, \end{array}$$

together with (7) and (8) and the definition of $S_{\ell }\left(t\right)$ (9), the proof of (17) is straightforward. With $\rho ={\hat{\psi }}_{\alpha }\left(\kappa \right)$, taking $a=\frac{\kappa }{\phi_{k}^{\prime }\left(t\right)}$ in (17), we have (18) □

**Proof of Theorem 1 (a).**  Let a∈S, i.e., $|\left(W_{\psi_{\alpha }}f\right)\left(t,a\right)|\geq \frac{\rho \mu }{2}$. Suppose $a\notin S_{\ell }$ for any *ℓ*, then

$$\begin{array}{c} |a-\frac{\kappa }{\phi_{\ell }^{\prime }\left(t\right)}|>\frac{\alpha \Delta }{\nu },\;\;\ell =1,\ldots ,K. \end{array}$$

Since supp$\left({\hat{\psi }}_{\alpha }\right)\subseteq \left[\kappa -\Delta \alpha ,\kappa +\Delta \alpha \right]$, we derive that

$$\begin{array}{c} |\left(Q_{\psi_{\alpha }}f\right)\left(t,a\right)\left|=\right|\sum_{\ell =1}^{K}A_{\ell }\left(t\right)e^{i2\pi \phi_{\ell }\left(t\right)}{\hat{\psi }}_{\alpha }\left(a\phi_{\ell }^{\prime }\left(t\right)\right)|=0. \end{array}$$

Furthermore,

$$\begin{array}{cc} |\left(W_{\psi_{\alpha }}f\right)\left(t,a\right)| & \leq |\left(W_{\psi_{\alpha }}f\right)\left(t,a\right)-\left(Q_{\psi_{\alpha }}f\right)\left(t,a\right)\left|+\right|\left(Q_{\psi_{\alpha }}f\right)\left(t,a\right)|\\ \; & \leq aMI_{1}\alpha +2\pi MBI_{2}a^{2}\alpha \\ \; & \leq \frac{\rho \mu }{2}. \end{array}$$

□

The last inequality follows (8) for α. This contradicts the condition that $|\left(W_{\psi_{\alpha }}f\right)\left(t,a\right)|\geq \frac{\rho \mu }{2}$. Consequently, there exists at least one *ℓ*, 1≤ℓ≤K, such that $a\in S_{\ell }$. Since $|a-\frac{\kappa }{\phi_{\ell }^{\prime }\left(t\right)}|\leq \frac{\alpha \Delta }{\nu }$ and $\min_{1\leq k\neq \ell \leq K}\left|\frac{1}{\phi_{\ell }^{\prime }\left(t\right)}-\frac{1}{\phi_{k}^{\prime }\left(t\right)}\right|=\eta >\frac{2\Delta \alpha }{\kappa \nu }$, *ℓ* is unique, i.e., $S_{\ell }\;\left(1\leq \ell \leq K\right)$ are disjoint. Then, we show that each $S_{\ell }$ is non-empty. To this regard, we prove that $\frac{\kappa }{\phi_{\ell }^{\prime }\left(t\right)}\in S$. Indeed, according to (18), we have

$$\begin{array}{c} |\left(Q_{\psi_{\alpha }}f\right)(t,\frac{\kappa }{\phi_{\ell }^{\prime }\left(t\right)}|=|\rho A_{\ell }\left(t\right)|\geq \rho \mu >\frac{3\mu \rho }{4}. \end{array}$$

It implies

$$\begin{array}{cc} |\left(W_{\psi_{\alpha }}f\right)\left(t,\frac{\kappa }{\phi_{\ell }^{\prime }\left(t\right)}\right| & \geq |\left(Q_{\psi_{\alpha }}f\right)\left(t,\frac{\kappa }{\phi_{\ell }^{\prime }\left(t\right)}\right)\left|-\right|\left(W_{\psi_{\alpha }}f\right)\left(t,\frac{\kappa }{\phi_{\ell }^{\prime }\left(t\right)}\right)-\left(Q_{\psi_{\alpha }}f\right)\left(t,\frac{\kappa }{\phi_{\ell }^{\prime }\left(t\right)}\right)|\\ \; & >\frac{3\mu \rho }{4}-\alpha \left(\kappa MI_{1}/\phi_{\ell }^{\prime }\left(t\right)+2\pi MBI_{2}\kappa^{2}/{\left(\phi_{\ell }^{\prime }\left(t\right)\right)}^{2}\right)\\ \; & >\frac{3\mu \rho }{4}-\alpha \left(\kappa MI_{1}/\nu +2\pi MBI_{2}\kappa^{2}/\nu^{2}\right)\\ \; & >\frac{3\mu \rho }{4}-\frac{\mu \rho }{4}\\ \; & =\frac{\mu \rho }{2}. \end{array}$$

Hence, $\frac{\kappa }{\phi_{\ell }^{\prime }\left(t\right)}\in S$.

**Proof of (10).**  By the definition of ${\overset{ˇ}{a}}_{\ell }$, we have

(19) $$\begin{array}{cc} |\left(W_{\psi_{\alpha }}f\right)\left(t,{\overset{ˇ}{a}}_{\ell }\right)| & \geq |\left(W_{\psi_{\alpha }}f\right)(t,\frac{\kappa }{\phi_{\ell }^{\prime }\left(t\right)}\left|\geq \right|\left(Q_{\psi_{\alpha }}f\right)\left(t,\frac{\kappa }{\phi_{\ell }^{\prime }\left(t\right)}\right)|\\ \; & -\alpha \left(\kappa MI_{1}/\left(\phi_{\ell }^{\prime }\left(t\right)\right)+2\pi MBI_{2}\kappa^{2}/{\left(\phi_{\ell }^{\prime }\left(t\right)\right)}^{2}\right)\\ \; & =\rho |A_{\ell }\left(t\right)|-\alpha \left(\kappa MI_{1}/\phi_{\ell }^{\prime }\left(t\right)+2\pi MBI_{2}\kappa^{2}/{\left(\phi_{\ell }^{\prime }\left(t\right)\right)}^{2}\right)\\ \; & \geq \rho |A_{\ell }\left(t\right)|-\alpha \left(\kappa MI_{1}/\nu +2\pi MBI_{2}\kappa^{2}/\nu^{2}\right). \end{array}$$

□

On the other hand, by (14), we have

(20) $$\begin{array}{cc} |\left(W_{\psi_{\alpha }}f\right)\left(t,{\overset{ˇ}{a}}_{\ell }\right)| & \leq |\left(Q_{\psi_{\alpha }}f\right)\left(t,{\overset{ˇ}{a}}_{\ell }\right)|+\alpha \left({\overset{ˇ}{a}}_{\ell }MI_{1}+2\pi MBI_{2}{\overset{ˇ}{a}}_{\ell }^{2}\right)\\ \; & =|A_{\ell }\left(t\right)e^{i2\pi \phi_{\ell }\left(t\right)}{\hat{\psi }}_{\alpha }\left({\overset{ˇ}{a}}_{\ell }\phi_{\ell }^{\prime }\left(t\right)\right)|+\alpha \left({\overset{ˇ}{a}}_{\ell }MI_{1}+2\pi MBI_{2}{\overset{ˇ}{a}}_{\ell }^{2}\right)\\ \; & \leq \rho |A_{\ell }\left(t\right)|+\alpha \left(\left(\alpha \Delta /\nu +\kappa /\phi_{\ell }^{\prime }\left(t\right)\right)MI_{1}+2\pi MBI_{2}{\left(\alpha \Delta /\nu +\kappa /\phi_{\ell }^{\prime }\left(t\right)\right)}^{2}\right)\\ \; & \;(\mathrm{by}\;|{\overset{ˇ}{a}}_{\ell }-\frac{\kappa }{\phi_{\ell }^{\prime }\left(t\right)}|\leq \frac{\alpha \Delta }{\nu })\\ \; & \leq \rho |A_{\ell }\left(t\right)|+\alpha^{3}2\pi MBI_{2}\Delta^{2}/\nu^{2}+\alpha^{2}\left(4\pi MBI_{2}\Delta \kappa /\nu^{2}+MI_{1}\Delta /\nu \right)\\ \; & +\alpha \left(MI_{1}\kappa /\nu +2\pi MBI_{2}\kappa^{2}/\nu^{2}\right), \end{array}$$

where the last inequality follows from (5). Combining (19) and (20) yields (10).

**Proof of (11).**  Let $\left(W_{\psi_{\alpha }}f\right)\left(t,{\overset{ˇ}{a}}_{\ell }\right)=\left|\left(W_{\psi_{\alpha }}f\right)\left(t,{\overset{ˇ}{a}}_{\ell }\right)\right|e^{i2\pi \theta_{\ell }\left(t\right)}$ for some real valued function $\theta_{\ell }\left(t\right)$. Note that for any complex number $z_{1}$, $z_{2}$, $\left|\right|z_{1}\left|-\right|z_{2}\left||\leq \right|z_{1}-z_{2}|$, we deduce that

$$\begin{array}{cc} \; & A_{\ell }\left(t\right)|{\hat{\psi }}_{\alpha }\left({\overset{ˇ}{a}}_{\ell }\phi_{k}^{\prime }\left(t\right)\right)-\rho |\leq A_{\ell }\left(t\right)\left|{\hat{\psi }}_{\alpha }\left({\overset{ˇ}{a}}_{\ell }\phi_{\ell }^{\prime }\left(t\right)\right)e^{i2\pi \phi_{\ell }\left(t\right)}-\rho e^{i2\pi \theta_{\ell }\left(t\right)}\right|\\ \; & =|{\hat{\psi }}_{\alpha }\left({\overset{ˇ}{a}}_{\ell }\phi_{\ell }^{\prime }\left(t\right)\right)A_{\ell }\left(t\right)e^{i2\pi \phi_{\ell }\left(t\right)}-\rho A_{\ell }\left(t\right)e^{i2\pi \theta_{\ell }\left(t\right)}|\\ \; & \leq |{\hat{\psi }}_{\alpha }\left({\overset{ˇ}{a}}_{\ell }\phi_{\ell }^{\prime }\left(t\right)\right)A_{\ell }\left(t\right)e^{i2\pi \phi_{\ell }\left(t\right)}-\left(W_{\psi_{\alpha }}f\right)\left(t,{\overset{ˇ}{a}}_{\ell }\right)\left|+\right|\left(W_{\psi_{\alpha }}f\right)\left(t,{\overset{ˇ}{a}}_{\ell }\right)-\rho A_{\ell }\left(t\right)e^{i2\pi \theta_{\ell }\left(t\right)}|\\ \; & \leq |{\hat{\psi }}_{\alpha }\left({\overset{ˇ}{a}}_{\ell }\phi_{\ell }^{\prime }\left(t\right)\right)A_{\ell }\left(t\right)e^{i2\pi \phi_{\ell }\left(t\right)}-\left(Q_{\psi_{\alpha }}f\right)\left(t,{\overset{ˇ}{a}}_{\ell }\right)\left|+\right|\left(Q_{\psi_{\alpha }}f\right)\left(t,{\overset{ˇ}{a}}_{\ell }\right)-\left(W_{\psi_{\alpha }}f\right)\left(t,{\overset{ˇ}{a}}_{\ell }\right)|\\ \; & +\left|\right|\left(W_{\psi_{\alpha }}f\right)\left(t,{\overset{ˇ}{a}}_{\ell }\right)\left|-\rho A_{\ell }\left(t\right)\right|\\ \; & \leq {\overset{ˇ}{a}}_{\ell }MI_{1}\alpha +2\pi MBI_{2}{\overset{ˇ}{a}}_{\ell }^{2}\alpha \\ \; & +\alpha^{3}2\pi MBI_{2}\Delta^{2}/\nu^{2}+\alpha^{2}\left(4\pi MBI_{2}\Delta \kappa /\nu^{2}+MI_{1}\Delta /\nu \right)+\alpha \left(MI_{1}\kappa /\nu +2\pi MBI_{2}\kappa^{2}/\nu^{2}\right)\\ \; & \leq \alpha^{3}4\pi MBI_{2}\Delta^{2}/\nu^{2}+\alpha^{2}\left(8\pi MBI_{2}\Delta \kappa /\nu^{2}+2MI_{1}\Delta /\nu \right)+\alpha \left(2MI_{1}\kappa /\nu +4\pi MBI_{2}\kappa^{2}/\nu^{2}\right), \end{array}$$

where the second last inequality follows from (17), (14) and (10), and the last inequality uses the fact ${\overset{ˇ}{a}}_{\ell }\leq \kappa /\nu +\Delta \alpha /\nu$. With ${\hat{\psi }}_{\alpha }\left({\overset{ˇ}{a}}_{\ell }\phi_{\ell }^{\prime }\left(t\right)\right)=\hat{\psi }\left(\frac{{\overset{ˇ}{a}}_{\ell }\phi_{\ell }^{\prime }\left(t\right)}{\alpha }\right)$ and $\rho =\hat{\psi }\left(\frac{\kappa }{\alpha }\right)$, for sufficiently small α, (11) holds by the property of continuity of $\hat{\psi }$. □

**Proof of (12).**  By (14), (15) and (17), we have

$$\begin{array}{cc} \; & |\left(W_{\psi_{\alpha }}f\right)\left(t,{\overset{ˇ}{a}}_{\ell }\right)-\rho f_{\ell }\left(t\right)|\\ \; & \leq |\left(W_{\psi_{\alpha }}f\right)\left(t,{\overset{ˇ}{a}}_{\ell }\right)-\left(Q_{\psi_{\alpha }}f\right)\left(t,{\overset{ˇ}{a}}_{\ell }\right)\left|+\right|\left(Q_{\psi_{\alpha }}f\right)\left(t,{\overset{ˇ}{a}}_{\ell }\right)-\left(Q_{\psi_{\alpha }}f\right)\left(t,\frac{\kappa }{\phi_{\ell }^{\prime }\left(t\right)}\right)|\\ \; & +|\left(Q_{\psi_{\alpha }}f\right)\left(t,\frac{\kappa }{\phi_{\ell }^{\prime }\left(t\right)}\right)-\rho f_{\ell }\left(t\right)|\\ \; & \leq {\overset{ˇ}{a}}_{\ell }MI_{1}\alpha +2\pi MBI_{2}{\overset{ˇ}{a}}_{\ell }^{2}\alpha +|\sum_{\ell =1}^{K}A_{\ell }\left(t\right)e^{i2\pi \phi_{\ell }\left(t\right)}\left|\right|{\hat{\psi }}_{\alpha }\left({\overset{ˇ}{a}}_{\ell }\phi_{\ell }^{\prime }\left(t\right)\right)-{\hat{\psi }}_{\alpha }\left(\kappa \right)|\\ \; & \leq \alpha^{3}2\pi MBI_{2}\Delta^{2}/\nu^{2}+\alpha^{2}\left(4\pi MBI_{2}\Delta \kappa /\nu^{2}+MI_{1}\Delta /\nu \right)+\alpha \left(MI_{1}\kappa /\nu +2\pi MBI_{2}\kappa^{2}/\nu^{2}\right)\\ \; & +M\int \alpha |\varphi \left(\alpha x\right)||e^{-i2\pi {\overset{ˇ}{a}}_{\ell }\phi_{\ell }^{\prime }\left(t\right)}-e^{-i2\pi \kappa }|dx\\ \; & \leq \alpha^{3}2\pi MBI_{2}\Delta^{2}/\nu^{2}+\alpha^{2}\left(4\pi MBI_{2}\Delta \kappa /\nu^{2}+MI_{1}\Delta /\nu \right)+\alpha \left(MI_{1}\kappa /\nu +2\pi MBI_{2}\kappa^{2}/\nu^{2}\right)\\ \; & +2\pi MBI_{0}\alpha o\left(1\right). \end{array}$$

□

Using the fact $|e^{iu}-e^{iu}\left|\leq |u-v\right|$, together with (11) and (5), we obtain the last inequality.

## 3. Experimentation and Examples

In this section, we demonstrate the effectiveness of CWTSSO by solving the inverse problem of recovering IFs and IMFs on several examples. First, we will present three artificial signals in synthetic experiments. In each synthetic experiment, we consider white noise with zero mean and variance $\sigma^{2}$. The Signal-to-Noise Ratio (SNR) is defined by

$$\begin{array}{c} \mathrm{SNR}[\mathrm{dB}]=10\log_{10}\frac{\mathrm{var}(f)}{\sigma^{2}}. \end{array}$$

The running time (as shown in Table 1) and the mean square error (MSE) are used as performance measures. The MSE is defined as follows

$$\begin{array}{c} \mathrm{MSE}=∥f-\tilde{f}{∥}_{2}^{2}/{∥f∥}_{2}^{2}, \end{array}$$

where *f* is the original signal and $\tilde{f}$ denotes the recovered signal. Since the choice of noise is random, we repeat each experiment 500 times. The accuracy of the reconstructed IMFs is measured by the normalized mean square error (NMSE), calculated by averaging the MSE of 500 independent trials. Then, we shift our attention to real data, i.e., a bat echolocation signal. The overall performances of CWTSSO are compared with CWTSST in terms of execution time and NMSE.

We must comment that the comparisons are far from complete. High-order synchrosqueezing transforms are not included because those high-order synchrosqueezing transforms are designed to achieve perfect performance for strongly amplitude-modulated and frequency-modulated (AM–FM) modes. However, in this paper, we focus on general signals with the modest frequency modulation hypothesis for the IMFs constituting the blind source signal. In fact, our method can also be extended to signals with strongly modulated modes using adaptive continuous wavelet transform with time-varying parameters [38].

**Figure 2:**
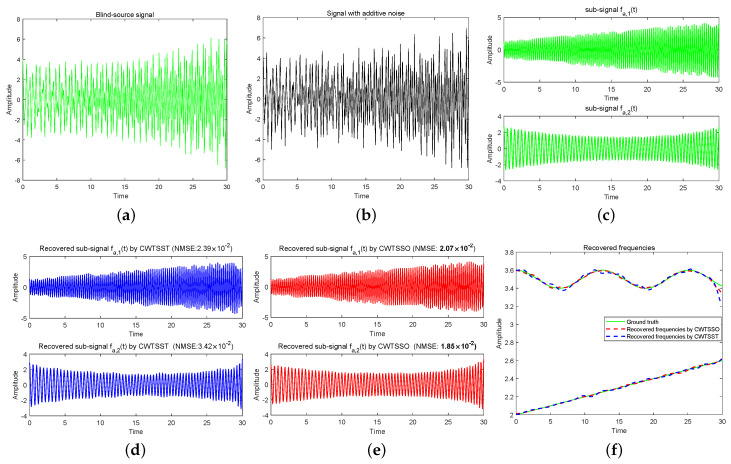
(**a**) Blind−source signal $f_{a}\left(t\right)$. (**b**) The observed signal $f_{a}\left(t\right)$ with additive noise (SNR = 20). (**c**) Sub-signals of blind source signal. (**d**) Recovered sub-signals by CWTSST. (**e**) Recovered sub-signals by CWTSSO. (**f**) Recovered frequencies.

**Example 1 (see Figure 2).**  *In the first example, we consider a blind source signal*

$$\begin{array}{c} f_{a}\left(t\right)=f_{a,1}\left(t\right)+f_{a,2}\left(t\right), \end{array}$$

*with an unknown number K=2 of unknown sub-signals*

$$\begin{array}{cc} \; & f_{a,1}\left(t\right)=\left(t/10+13/10\right)\cos\left(2\pi \left(7t/2+\sin\left(t/2\right)/5\right)\right)\;\;0<t<30,\\ \; & f_{a,2}\left(t\right)=\exp\left({\left(1-t/30\right)}^{2}+{\left(t/30\right)}^{3}\right)\cos\left(2\pi \left(2t+t^{2}/100\right)\right)\;\;0<t<30. \end{array}$$

*$f_{a}\left(t\right)$ is sampled uniformly with 512 sample points. In Figure 2, we plot the original signal $f_{a}\left(t\right)$ and the observed signal (SNR = 20 dB), together with the reconstructed results of sub-signals by CWTSST and CWTSSO. The comparisons of NMSE of recovered sub-signals show that CWTSSO delivers better performance than CWTSST. It also can be seen that the differences between the recovered frequencies and the truth by CWTSSO are smaller than ones by CWTSST. In estimating the running time, CWTSST takes much time since it needs to calculate synchrosqueezed transform and CWTSSO provides a better speed performance than CWTSST.*

**Figure 3:**
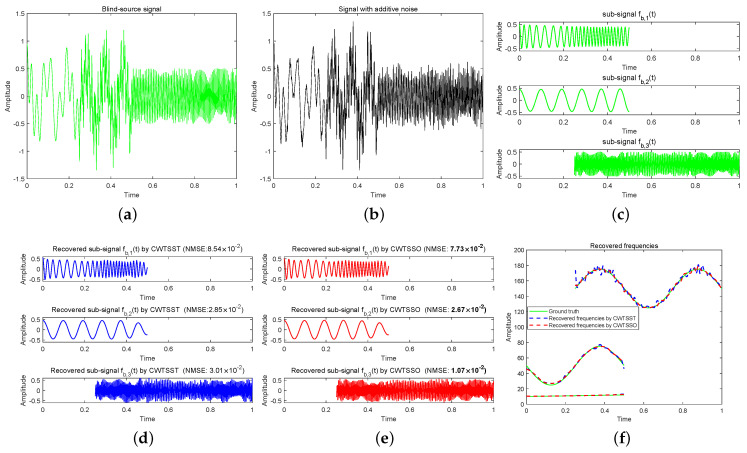
(**a**) Blind−source signal $f_{b}\left(t\right)$. (**b**) The observed signal $f_{b}\left(t\right)$ with additive noise (SNR = 15). (**c**) Sub-signals of blind source signal. (**d**) Recovered sub-signals by CWTSST. (**e**) Recovered sub-signals by CWTSSO. (**f**) Recovered frequencies.

**Example 2 (see Figure 3).**  *Next, we consider a challenging example, defined by*

$$\begin{array}{c} f_{b}\left(t\right)=f_{b,1}\left(t\right)+f_{b,2}\left(t\right)+f_{b,3}\left(t\right), \end{array}$$

*where*

$$\begin{array}{cc} \; & f_{b,1}\left(t\right)=\left(2t^{2}/5-2t/5+1/2\right)\cos\left(2\pi \left(50t+2\cos\left(4\pi t\right)\right)\right),0<t<1/2,\\ \; & f_{b,2}\left(t\right)=\left(t/20+9/20\right)\cos\left(2\pi \left(10t+2t^{2}\right)\right),0<t<1/2,\\ \; & f_{b,3}\left(t\right)=\left(1/2\right)\exp\left(-{\left(t-1/2\right)}^{2}/50\right)\cos\left(2\pi \left(150t+2\cos\left(4\pi t\right)\right)\right),1/4<t<1, \end{array}$$

And add a noise with SNR =15 dB to this signal. In Figure 3a,b, we display the blind source signal $f_{b}\left(t\right)$ and the observed signal with additive noise (SNR = 15). In this experiment, the sampling rate is 512 Hz. The results of the recovered sub-signals together with the recovery accuracy for both CWTSST and CWTSSO are depicted in Figure 3d,e. While the waveforms can be successfully recovered by CWTSST and CWTSSO, CWTSSO achieves a more accurate reconstruction. It can be verified by recovered frequencies, as shown in Figure 3f. Referring to running time, CWTSSO is more than seven orders-of-magnitude faster than that of CWTSST. This experiment suggests that CWTSSO has an obvious advantageous ability of efficiency and accuracy than CWTSST.

**Figure 4:**
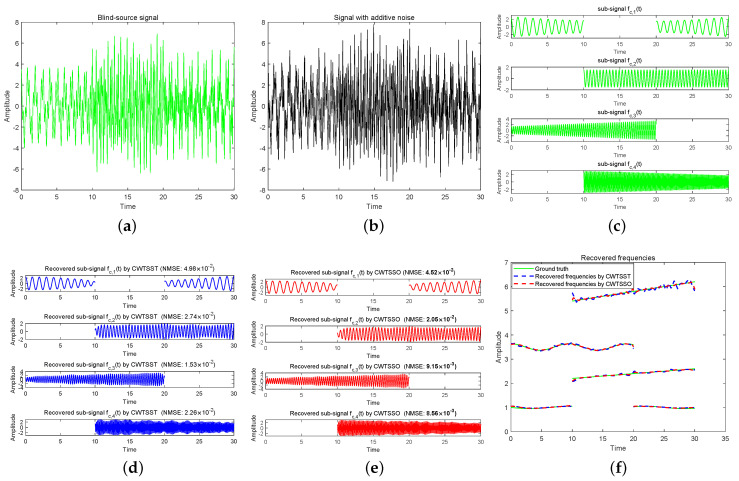
(**a**) Blind−source signal $f_{c}\left(t\right)$. (**b**) The observed signal $f_{c}\left(t\right)$ with additive noise (SNR = 10). (**c**) Sub-signals of blind source signal. (**d**) Recovered sub-signals by CWTSST. (**e**) Recovered sub-signals by CWTSSO. (**f**) Recovered frequencies.

**Example 3 (see Figure 4).**  *In this example, we illustrate the proposed method when applied to a non-stationary signal, i.e., a signal where amplitudes are also time-dependent in addition to frequencies, defined by*

$$\begin{array}{c} f_{c}\left(t\right)=f_{c,1}\left(t\right)+f_{c,2}\left(t\right)+f_{c,3}\left(t\right)+f_{c,4}\left(t\right), \end{array}$$

*where*

$$\begin{array}{cc} \; & f_{c,1}\left(t\right)=\exp\left({\left(1-t/30\right)}^{2}+{\left(t/30\right)}^{3}\right)\cos\left(2\pi \left(t+\cos\left(t/2\right)/10\right)\right),0<t<10\;\;\mathrm{and}\;\;20<t<30,\\ \; & f_{c,2}\left(t\right)=\left(3/2\right)\cos\left(2\pi \left(2t+t^{2}/100\right)\right),10<t<30,\\ \; & f_{c,3}\left(t\right)=\left(t/10+13/10\right)\cos\left(2\pi \left(7t/2+\sin\left(2t/3\right)/5\right)\right),0<t<20,\\ \; & f_{c,4}\left(t\right)=\left(2+\cos\left(2\pi t/100\right)\right)\cos\left(2\pi \left(5t+t^{2}/50\right)\right),10<t<30. \end{array}$$

The signal $f_{c}\left(t\right)$ is added with a white noise with SNR = 10 dB and sampled at a rate of 64 Hz on [0,30]. The results measuring the accuracy of mode reconstruction in terms of NMSE are shown in Figure 4d,e, with running times in Table 1. As shown, CWTSSO behaves better than CWTSST in each case, and CWTSSO is much faster than CWTSST. Figure 4f shows that instantaneous frequencies of both components are better extracted by CWTSSO than those by CWTSST.

**Figure 5:**
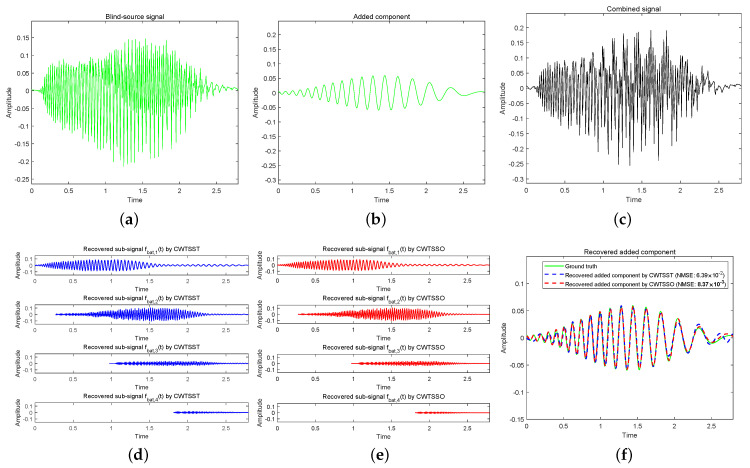
(**a**) A bat echolocation signal $f_{\mathrm{bat}}$. (**b**) Added component $f_{\mathrm{bat},5}$. (**c**) Combined blind source signal $f_{\mathrm{bat}}+f_{\mathrm{bat},5}$. (**d**) Recovered sub-signals by CWTSST. (**e**) Recovered sub-signals by CWTSSO. (**f**) Recovered sub-signal $f_{\mathrm{bat},5}\left(t\right)$.

**Example 4 (see Figure 5).**  *We consider a real-word signal, namely, “a bat echolocation signal” $f_{\mathrm{bat}}$ emitted by a large brown bat, and discover that it consists of four IMFs,*

$$\begin{array}{c} f_{\mathrm{bat}}=f_{\mathrm{bat},1}+f_{\mathrm{bat},2}+f_{\mathrm{bat},3}+f_{\mathrm{bat},4}. \end{array}$$

*To convince ourselves that this decomposition makes sense, we add an unknown signal $f_{\mathrm{bat},5}$, given by*

$$\begin{array}{c} f_{\mathrm{bat},5}\left(t\right)=\left(4/125-\left(7/254\right)\cos\left(2\pi t\right)\right)\cos\left(2\pi \left(30t-13t^{2}\right)\right), \end{array}$$

To $f_{\mathrm{bat}}$ and decompose the combined blind source signal $f_{\mathrm{bat}}+f_{\mathrm{bat},5}$, and discover that it consists of five IMFs. Both CWTSSO and CWTSST can successfully recover the IMFs, while the MSE of the added component $f_{\mathrm{bat},5}\left(t\right)$ by CWTSSO and CWTSST are $8.37\times 10^{-3}$ and $6.39\times 10^{-2}$, respectively. Table 1 shows the execution-time comparison. Through the comparison, it demonstrates that CWTSSO is superior to CWTSST in both efficiency and effectiveness.

## 4. Conclusions

In this paper, we develop a rigorous mathematical theory of signal separation operator based on continuous wavelet transform (CWTSSO). An in-depth error analysis study of instantaneous frequency estimation and component recovery is provided. In contrast to CWTSST, CWTSSO is a direct method for mode decomposition and retrieval by plugging the extracted instantaneous frequency values in the signal separation operator. Hence, the CWTSSO provides superior efficiency against the CWTSST. Numerical experiments demonstrate the efficiency and effectiveness of CWTSSO for a blind source non-stationary signal separation. A Matlab implementation of CWTSSO can be obtained by communicating with authors. Our future work will focus on algorithms that apply the proposed scheme to engineering and scientific fields, such as mobile communication, sensor arrays, radar systems. 

## Figures and Tables

**Table 1:** Comparisons of running time(s).

	Example 1	Example 2	Example 3	Example 4
CWTSST	15.46	28.62	133.27	13.81
CWTSSO	**2.78**	**3.87**	**17.77**	**1.94**

## Data Availability

The raw data supporting the conclusions of this article will be made available by the authors on request.
